# Effect of autoclaving process on trueness and qualitative marginal fit of intraoral scan body manufactured from two materials: An in vitro study

**DOI:** 10.1371/journal.pone.0325068

**Published:** 2025-06-23

**Authors:** Fábio Henrique de Paulo Costa Santos, Patrícia Santos de Melo, Paulo Sérgio Borella, Flávio Domingues das Neves, Karla Zancope

**Affiliations:** 1 Department of Occlusion, Fixed Prosthodontics, and Dental Materials, School of Dentistry, Federal University of Uberlandia, Uberlandia, Minas Gerais, Brazil; 2 Department of General Practice, School of Dentistry, Virginia Commonwealth University, Richmond, Virginia, United States of America; University of Vigo, SPAIN

## Abstract

**Statement of the problem:**

It is not clear the effect of multiple autoclaving processes on the accuracy of scan bodies. Autoclaving dental disposables enables reuse, reducing the environmental impact of raw materials and waste.

**Purpose:**

This in vitro laboratory study evaluated the effect of the autoclaving process on the intraoral scan body (SB) manufactured from different materials (Titanium and PEEK), positioned at the implant and abutment levels.

**Methods:**

Two models, each with 10 implants, were created to evaluate scan body trueness made of two materials (Titanium and PEEK), and implant junction positioned at two levels (implant and abutment levels). Analyses were conducted at five distinct time points: T0 (control group with new SB), T1 (after one autoclaving cycle), T2 (10 cycles), T3 (50 cycles), and T4 (100 cycles). Forty SBs were divided into four groups (n = 10), fixed on the models according to the initial positioning, using the recommended torque, and scanned 10 times using an intraoral Sccaner (InEOS X5 scanner, Dentsply Sirona). The surface deviation was evaluated by .STL mesh overlay using an image software (Geomagic Control X software). Microscopic analyses of marginal adaptation were performed simultaneously with the digital analysis using an optical microscope (Mitutoyo™-500 Optical Microscope) with 40x magnification at four places (mesial, distal, buccal, and lingual). Statistical analysis was performed using statistical software (SPSS 29.0 software), with α = 0,05. Data were subjected to the Shapiro-Wilk normality test. To assess spatial variation after the cycles, Repeated Measures ANOVA with Bonferroni correction was used. For microscopic qualitative analysis, percentile was used to check the prevalence of clinically adapted and misfitting faces.

**Results:**

Statistically significant differences in mesh deformation were detected, with PEEK SB showing greater deformation compared to Titanium, especially at the abutment level, after 100 cycles (P < 0.01). Microscopic analysis revealed that 100% of the faces were classified as “Clinically Adapted” by the three calibrated evaluators (KAPPA > 0.08).

**Conclusions:**

Titanium scan bodies showed less surface deviation than PEEK but both remained below 50 µm. Internal-junction scan bodies were more affected than abutment-level ones. All scan bodies stayed within acceptable limits and viable after 100 autoclaving cycles.

## Introduction

An important step in fabricating implant prostheses using digital workflows is implant acquisition through intraoral scanning CAI (Computer-Aided Image) [1]. Those digital impressions capture the scan body and transfer the position of the implant from the mouth to a digital environment, ensuring that the STL (Standard Tessellation Language) file obtained virtually is accurate to the scanned clinical situation [2]. The trueness and dimensional stability of the scan body (SB) is crucial for the success of the procedure, as any alteration in their surface or geometry can cause variations in the digital model and consequently compromise the adaptation and feasibility of the works planned in CAD (Computer-Aided Design) software [3–5].

Blood and saliva commonly contaminate the surface of SB during the clinical workflow when taking digital impressions for fabricating implant-supported restorations. Thus, efficient decontamination is necessary to reuse them [6]. Decontamination is an essential practice for patient safety and infection prevention, commonly used in dental offices to ensure a safer working environment [7]. Although autoclaving is important, there are still uncertainties about its influence on the integrity and accuracy of these SB after consecutive autoclaving process [8].

Today, there are various types of SB available on the market. These can be made from several materials, such as PEEK (Polyether-Ether-Ketone) and Titanium. They are designed to be screw-retained on both abutments and implants. PEEK is a high-performance thermoplastic material that is resistant and biocompatible. Its optical properties help improve the quality of images scanned by reducing light reflections. It also presents good cost-effectiveness. It is recommended to exercise caution when using it, as its dimensions may change when exposed to heat [9]. This characteristic leads many manufacturers to contraindicate autoclaving. Titanium’s SB is a new option to solve this problem. Made of a metallic alloy, it allows for disinfection by autoclaving and is radiopaque, enabling radiographic verification of the marginal fit. However, its use as a material for SB production is relatively new and presents cost-related challenges and light reflection [10].

Furthermore, the influence of autoclaving on dental disposable items is a highly relevant topic in modern dentistry. Reusing these items without damaging the transfer component would reduce the environmental impact, both in terms of raw material acquisition and in minimizing the disposal of viable transfer items for use [11]. The effect of the sterilization process on the surface and adaptation of SBs is uncertain and presents a gap in the literature that needs to be better elucidated. The null hypothesis stated that the sterilization process would not affect the surface deviation of the SBs or the marginal fit at their junction. This study aims to evaluate the influence of the autoclaving process on the surface deviation trueness and marginal adaptation of SBs made from two different materials, at two different prosthetic junctions (implant level and abutment level).

## Materials and methods

**Fig 1:**
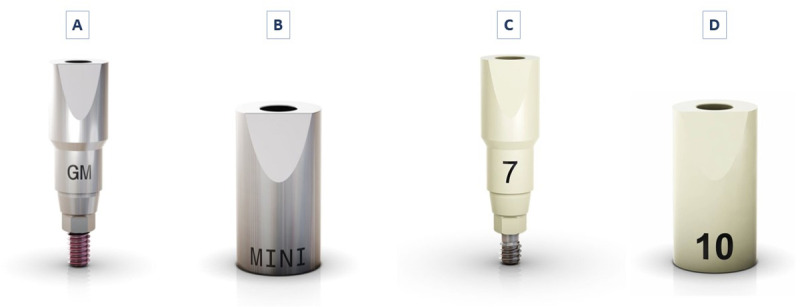
Scan bodies. Titanium SBs implant level (GMT), abutment level (MPT). PEEK scan bodies implant level (GMP), and abutment level (MPP).

This in vitro laboratory study consists of four groups (n=10). Two models, each containing 10 implant analogs (GM, Neodent, Curitiba, Brazil) were used to install SBs (GM, Neodent, Curitiba, Brazil), made of two materials (PEEK and Titanium), placed at two different levels: the implant analog level and the Multi-Unit Abutment (Neodent, Curitiba, Brazil) level (Fig 1).

### Master model and positioning guide production

**Fig 2:**
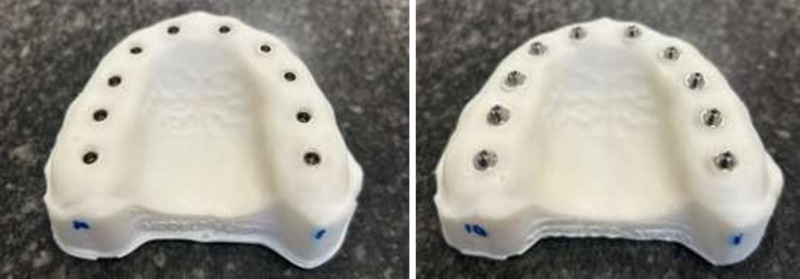
Master model. Printed models with 10 analogs positioned (A- implant level; B- abutment level).

Two printed models were created using CAD software (Exocad 3.2 Elefsina, Darmstadt, Germany). Each model had 10 hybrid analogs placed in the slots, evenly distributed with the same distance and inclination, 0.5mm above the gingiva. Each model is specific to one type of analog: 1- Implant-level analog and 2- Abutment-level analog (Fig 2).

The repositioning guides were designed across the arch with intaglios capturing the chamfer shape of the SBs using open-source software (Meshmixer 3.5) based on the initial scan with the SBs in position. This guide was used throughout all analysis periods to reproduce the initial position of the SBs. All the objects were manufactured using an open-source, MSLA (Masked Stereolithography) 3D printer (Photon Mono M5s printer, Anycubic, Shenzhen Anycubic Technology Co.), which employs UV light with a wavelength of 405nm for precise material curing. The Photon Mono M5s printer’s print volume of 223.78 x 126.38 x 200 mm (XYZ) allowed the production of the components with high precision, while the 10.1-inch monochrome Liquid-crystal display (LCD), with a resolution of 13312x5120px (14K), ensured sharp and fine detail reproduction. A 3D printing resin (Prisma 3D Model 2.0 resin, Makertech Labs, São Paulo, Brazil) was employed to create the objects. The parameters used for the resin are presented in Table 1.

**Table 1:** Parameters used for printing the models and repositioning guide.

Layer Height (mm)	Botton Layer Count (s)	Exposure Time (s)	Botton Exposure Time (s)	Lifting/Retract Exposure (mm/min)
**0,05**	5	2,5	20	360

After printing, the models and positioning guides were washed in 99.8% isopropyl alcohol (CR CLEAN, São Paulo, Brazil), dried for 5 minutes, and post-cured for 30 minutes in the chamber (Anycubic, Shenzhen Anycubic Technology Co.). The analogs were fixed to the model using cyanoacrylate (Wurth, São Paulo, Brazil) and were numbered to identify their corresponding SB.

### Sterilization process and scanning

**Fig 3:**
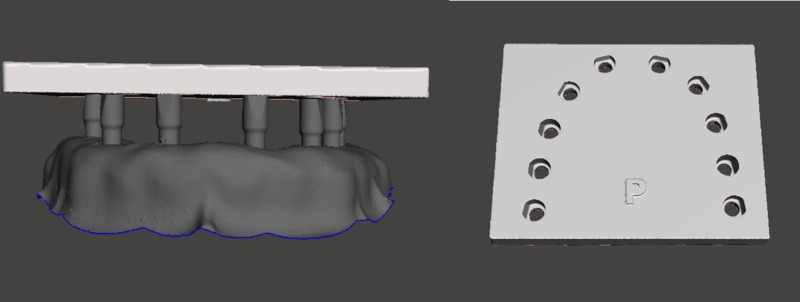
Reposition guide. Planned positioning guide in Meshmixer® software.

The SBs were screwed on their respective models with a torque of 10N using a dynamometer (Neodent, Curitiba, Brazil). After the different autoclaving cycles, the SBs were always brought to the same position, allowing, after scanning, to overlay STL files accurately, without positional variation in the chamfer region (Fig 3). The process started with the placement of the SBs on the models straight from the manufacturer’s package (T0).

The SBs were then unscrewed and packaged in sterilization pouches (Sterilex, São Paulo, Brasil) for the first sterilization cycle. The autoclaving cycles were performed in the autoclave (Bioclave - Dabi Atlante, Minas Gerais, Brazil – Class B) for forty minutes, at a maximum temperature of 134°C, followed by 20 minutes for cooling. The analysis periods were conducted at 5 distinct time points: T0 (control group with new SBs), T1 (after one autoclaving cycle), T2 (10 cycles), T3 (50 cycles), and T4 (100 cycles).

After the different sequential autoclaving cycles, they were positioned in their respective positions, always in the same position with the manufacturer’s recommended torque, 10 N/cm^2^. These models were taken to a calibrated bench scanner (InEOS X5, Dentsply Sirona) and scanned 10 times for each analysis period. Thus, at the end of the experiment, 200 STL files were obtained, allowing the evaluation of the effect of different autoclaving cycles on the SBs. The STL files from the scans were placed in a folder with specific group and time identifiers for data organization.

### Mesh alignment and measurement

**Fig 4:**
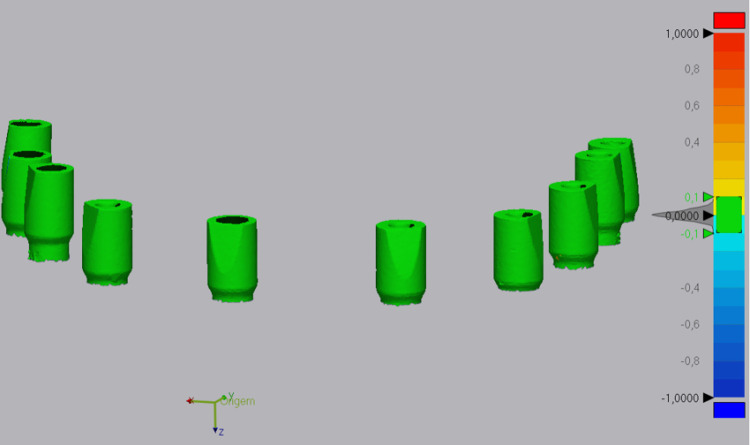
Alignment. Overlap of STL meshes from test group with control group using Geomagic Control software.

The surface deviation of the SBs after the autoclaving cycles was verified according to ISO 5275-4 (2020) for trueness. The 200 STL files generated by the scans were initially aligned in the alignment tool using metrology software (Geomagic Control) to match each file with the control group in a 1:1 ratio (Fig 4) [12]. The “best fit” alignment tool was applied after cropping all areas below the junction of the SBs to eliminate potential discrepancies from other regions, including the screw channel area. This software is designed to analyze 3D volumetric discrepancies and provides the root mean square (RMS) deviation values between the control and test groups. Values close to 0 indicate minimal surface deviations, shown in green on the color map. Maximum positive deviations, close to 1, are represented in red, while maximum negative deviations, close to −1, are shown in blue.

### Marginal fit

For the qualitative assessment, image analysis was performed using an optical microscope (Mitutoyo TM-500) at 40x magnification after each cycle, completing five analyses (T0–T4). The samples were secured on two analogs, each positioned individually on cubic bases (implant level and abutment level), with sides numbered from 1 to 4 representing the buccal, lingual, mesial, and distal surfaces, respectively. These bases were then placed under the optical microscope to measure the marginal misfits at the SB/implant and SB/abutment junctions, ensuring consistent measurement in the same area across all analysis time points. The images of the margins of the SBs were recorded for each face (buccal, lingual, mesial, and distal). This way, 800 measurements were taken. The visual discrepancies between the SB and analog were classified as clinically adapted, misfitting, or highly misfitting based on the subjective evaluation of the margins by three trained evaluators. A quantitative analysis was performed based on the classification of the three evaluators, where “clinically adapted” was considered a correct fit if there was no observable gap between the SB and the analog [13].

### Statistical analysis

Statistical analysis was performed using SPSS 29.0 software. Data were subjected to the Shapiro-Wilk normality test to determine the distribution pattern. One-way ANOVA followed by Tukey was performed to assess the differences between the groups within each cycle time (t0, t1, t2, t3, t4). Repeated Measures ANOVA with Bonferroni correction was used to assess surface deviation analysis of the SBs after the cycles (t0 to t4), considering α=0,05. For the qualitative microscopic analysis, Percentile analysis was performed through the Kaplan-Meier test to determine the prevalence of clinically adapted and misfitting faces [14].

## Results

### Surface deviation

The mean and standard deviation for the surface deviation in different groups are presented in Table 2. Statistically significant differences were observed for different materials at the different junctions for all groups in t1 (p < 0.05), the GMT group showed the lowest deviation. In t2, GMP and MPP did not present differences (p = .0736), the GMT group showed the lowest deviation. In t3, both GMP and MPP (p = .331), and MPT and GMT (p = .439) did not present differences. Similar results were found in t4, MPT and GMT were similar (p = 0.534) in this time also. The surface deviation across different times also presented statistical differences (p < 0.001). The groups GMT (p < 0.001), GMP (p < 0.001), MPP (p > 0.001), and MPT (p > 0.001) showed the highest values of deviation within t4 when compared to t1.

**Table 2:** Mean values of surface deviation (µm) ± standard deviation.

		Time baseline			
		t1	t2	t3	t4
**TI**	**GMT**	1.7 ± 1.2 Aa	6.7 ± 5.1 Aa	14.7 ± 3.9 Ab	18.4 ± 3.4 Ac
	**MPT**	8.5 ± 2.2 Ba	15.4 ± 3.9 Bb	17.7 ± 3.2 Ab	21.0 ± 2.5 Ab
**PEEK**	**GMP**	16.2 ± 4.9 Ca	25.5 ± 4.4 Cb	37.6 ± 6.1 Bc	49.6 ± 6.4 Cd
	**MPP**	20.6 ± 3.6 Da	27.8 ± 5.2 Cb	34.2 ± 2.8 Bc	41.0 ± 1.7 Bd

Uppercase letters indicate significant differences between lines. Lowercase letters indicate significant difference between rows.

### Marginal fit

**Fig 5:**
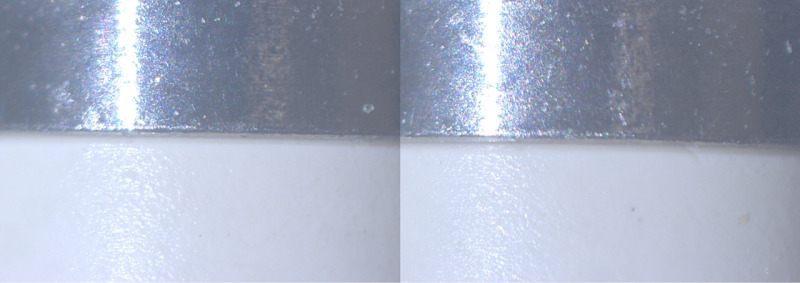
Fit. GMP scan bodies marginal adaptation after different sterilization cycles: A-T0; B- T4.

After analyzing the data, no differences were detected between the evaluated groups. At every cycle, the SBs were classified as “Adapted.” Even with a 40x magnification, it was not possible to detect changes in the marginal adaptation of the SB made from different materials and positioned at different prosthetic junctions (Fig 5). The 800 faces, considering all the cycles (T0 to T4), were evaluated by three calibrated assessors, and 100% of the faces were classified as “Adapted” (Kappa > 0.08).

## Discussion

The autoclaving process influenced the surface deviation but did not affect the marginal adaptation of SBs made from two different materials at two different prosthetic junctions. Thus, the null hypothesis was rejected.

Surface analysis using metrology software revealed discrepancies between the reference and test meshes and it may be a good choice to perform studies such as this one, as previously used in other studies [3,6,8]. The bench scanner, considered the most accurate device currently available, provided reliable reference models, and it has been reported in previous studies [15]. According to the results provided, the SBs made of PEEK exhibited greater surface deviation compared to Titanium SBs, showing increased deviation when attached to the implant. These findings suggest that the deformation may be related to either the repetitive process of screwing and unscrewing the SBs, which could cause compression-related deformation when torque is applied, or to multiple heat cycles from the sterilization process [16]. Further studies are needed to investigate this issue.

Sequential cycles led to surface deviations across all groups compared to the initial condition. Among them, the GMP group exhibited the most significant variation after 100 cycles, followed by MPP, GMT, and MPT. In this study, the sterilization process was conducted according to the manufacturer’s instructions in a calibrated machine. Therefore, the results may vary depending on deviations in the autoclaving process from the recommended procedure. [17].

As observed, even after 100 autoclave cycles, the SBs remained clinically adapted and suitable for use. The surface deformation is within the acceptable limit for precise alignment in CAD software (Fig 4). Therefore, the findings of this study could suggest that it is safe to promote a disinfection protocol without interfering with the surface or integrity of the SB and contribute to reducing the disposal of SBs. This, in turn, would minimize the environmental impact caused by the excessive disposal of components that could be safely reused without compromising clinical outcomes.

This study used two commercially available options: SBs that can be screwed in either on the abutment or straight from the implant’s platform made of PEEK (Polyetheretherketone) and Titanium. Given the difficulties observed with PEEK, the Titanium SB was created to allow autoclaving and radiopaque, enabling radiographic adaptation analysis. However, its higher light reflection complicates scanning, which may influence the accuracy of the generated model Additionally, the cost of obtaining the raw material and the environmental impact associated with its procurement are significantly higher compared to PEEK [9,10].

According to the observed results, it can be suggested that the PEEK SBs groups deformed significantly more than those made of Titanium, especially after 50 cycles of sterilization (t3). This deformation becomes more pronounced after 100 cycles, though with values below 50 micrometers, which are clinically indistinguishable. Titanium SBs also exhibited surface variation, but with values around 20 micrometers, proving more stable for withstanding sequential autoclaving cycles, followed by tightening and loosening of screws.

Another interesting finding was that the implant-level SB was more susceptible to surface deviation when the prosthetic junction was evaluated. Their more accurate internal indexing has less tolerance for small variations, making them the group with the highest risk of mispositioning [16]. Based on this analysis, GMP (PEEK implant-level) was the group with the greatest deviation. On the other hand, GMT (Titanium abutment level) showed the lowest deviation. This might suggest that its index can influence the deviation. Abutment-level SBs had varying results depending on the material, with PEEK again showing greater variation, with values approaching 40 micrometers after 100 autoclaving cycles.

The surface deviation results corroborated what was observed in the microscopic analysis. PEEK and Titanium SBs showed clinically imperceptible misfit, as the qualitative analysis revealed that all 800 faces were classified as adapted even at 40x magnification. Thus, all components, regardless of material and prosthetic junction, even after 100 autoclaving cycles, did not exhibit deformations sufficient to warrant disposal or render them unsuitable for clinical use.

This work was done to align with the 12 Sustainable Development Goals of the World Health Organization (WHO) [11]. The 12 Sustainable Development Goals (SDGs) mentioned by the WHO are part of a global effort to promote well-being and sustainability worldwide. Evaluating the effect of the autoclaving process and knowing that it is possible to reuse SBs safely and without contamination significantly reduces the environmental impact, both during the raw material acquisition phase and in the mass disposal of SBs.

## Conclusion

Within the limitations of this comparative in vitro study, the titanium scan bodies used for intraoral scanning showed less surface deviation compared to PEEK scan bodies. Both materials had deviation values below 50 µm. Internal-junction scan bodies were more susceptible to surface deviations than abutment-level scan bodies. Despite the observed surface deviations, all tested scan bodies remained within acceptable limits and were considered viable after 100 autoclaving cycles. These findings may support more evidence-based clinical decisions regarding the selection of scan body materials and their reuse after sterilization, promoting biologically safer, cost-effective, and environmentally sustainable digital impressions.
